# Using biomarker signature patterns for an mRNA molecular diagnostic of mouse embryonic stem cell differentiation state

**DOI:** 10.1186/1471-2164-8-210

**Published:** 2007-07-03

**Authors:** Daniel YL Yap, David K Smith, Xue W Zhang, Jeffrey Hill

**Affiliations:** 1 Bioinformatics Institute, 30 Biopolis Street, #07-01, Matrix, 138671, Singapore; 2 Department of Biochemistry, Li Ka Shing Faculty of Medicine, The University of Hong Kong, 21 Sassoon Road, Pokfulam, Hong Kong SAR, China; 3 College of Light Industry and Food Sciences, South China University of Technology, Guangzhou, Guangdong, China

## Abstract

**Background:**

The pluripotency and self-renewal capabilities, which define the "stemness" state, of mouse embryonic stem (ES) cells, are usually investigated by functional assays or quantitative measurements of the expression levels of known ES cell markers. Strong correlations between these expression levels and functional assays, particularly at the early stage of cell differentiation, have usually not been observed. An effective molecular diagnostic to properly identify the differentiation state of mouse ES cells, prior to further experimentation, is needed.

**Results:**

A novel molecular pattern recognition procedure has been developed to diagnose the differentiation state of ES cells. This is based on mRNA transcript levels of genes differentially expressed between ES cells and their differentiating progeny. Large publicly available ES cell data sets from various platforms were used to develop and test the diagnostic model. Signature patterns consisting of five gene expression levels achieved high accuracy at determining the cell state (sensitivity and specificity > 97%).

**Conclusion:**

The effective ES cell state diagnostic scheme described here can be implemented easily to assist researchers in identifying the differentiation state of their cultures. It also provides a step towards standardization of experiments relying on cells being in the stem cell or differentiating state.

## Background

Embryonic stem (ES) cells, which are derived from the inner cell mass (ICM) of the blastocyst stage of an embryo, are characterized by their ability to self-renew and to produce progeny that can give rise to the three germ layers (ectoderm, mesoderm and endoderm) [1]. Growth and sustenance of these cells *in vitro *requires the presence of mouse embryonic fibroblast (MEF) cells as feeder layers [2]. These supportive feeder layers consist of connective tissue cells that form the matrix upon which ES cells grow. Recently, feeder-free systems have also been developed to culture ES cells [3,4]. Assuming that cells are cultured on correct medium with sufficient amounts of secreted factors, such as the cytokine leukemia inhibitory factor (LIF), several key transcription factors work together to activate or inhibit target genes to maintain ES cells in a proliferative, non-differentiating state. For example, transcription factors such as Nanog [5,6], Oct-4 [7,8], Sox2 [9,10], and Sall4 [11,12] have helped scientists elucidate how embryonic stem cells replicate without differentiating.

To attempt to identify the full set of genes involved in maintaining the stem cell state ("stemness"), high-throughput technologies like microarrays [13], chromatin immuno-precipitation [6] and MPSS [14] have been used to compare gene expression profiles between ES cells and differentiating cells. These techniques allow the detection of differentially expressed genes, even at low transcript levels, but with varying levels of sensitivity. However, these studies can be compromised if the ES cell sample contains a mixture of cells with depleted populations remaining in the stem cell state. This may lead to many markers, especially those expressed at low levels, not being detected [15]. Alternatively, the differentiated cell sample may have reached a lineage specific differentiation stage where many of the genes expressed are specific to the developmental process (early organogenesis) and not relevant to the initiation of differentiation. Determining the time point for genome-wide comparative analyses of these cell populations is critical to derive a consistent set of "stemness" genes.

Functional assays (e.g. chimeric mice, embryoid body generation, colony forming potential) or quantitative RT-PCR measurements on known ES cell gene markers are commonly used to determine the timeline for the loss of pluripotency [16]. However, the relationship between gene expression and loss of pluripotency is complex [17]. Two ES cell markers (SSEA-1, Oct-4) have been found to show no unequivocal temporal correlation between the expression of the genes and the loss of pluripotency [18]. The differentiation potential and self-renewal capability of the ES cell colonies decreased rapidly during the initial 40 hours after LIF removal, yet the expression level of ES cell markers remained relatively unaltered for up to 80 hours [18]. In another study, six ES cell markers (Oct-4, Rex1, Gbx2, Nanog, FoxD3 and Sox2) were found to decrease in expression level at variable time points and rates [13]. Glover et al. [19] used a meta-analysis to identify a small set of genes that show consistent changes in expression upon cell differentiation, giving a unique ES cell signature. However, they also showed that the total of genes in a signature depended on the protocol used. These studies suggest that studies of single ES cell markers with functional assays are not sufficient to define what is required to maintain the stem cell state. Several factors need to be balanced in a particular way for ES cells to remain in a self-renewing state. If this balance shifts, ES cells may begin to differentiate [20].

Here, we devised a novel molecular pattern recognition procedure for the diagnosis of the ES cell state, based on the expression patterns of a group of genes. Genes that are differentially expressed between ES cells and differentiating cells were identified from large publicly available ES cell data sets. A diagnostic signature pattern consisting of five biomarkers was developed and assessed using a cross-validation strategy to give high sensitivity and specificity. These biomarkers, which are also expressed at low levels in the feeder layers of mouse embryonic fibroblast (MEF) cells, can be used to determine experimentally the differentiation state of ES cell cultures. Therefore they can also provide a step towards standardization of ES cell studies by identifying definitively their closest embryological equivalents. This development is a step in the direction that reduces the possibility of signal contamination from the feeder layer of MEF cells. Use of a subset of differentially expressed genes, which encode secreted or transmembrane proteins, may allow development of a non-destructive proteomic assay to determine the state of an ES cell culture.

## Results

### Identifying genes that correlate with maintenance or loss of pluripotency in ES cells

114 genes were found to meet the conditions (described in the Methods section) to be considered differentially expressed between embryonic stem cells and their differentiating progenies after LIF removal and so form G_stemness _(see Additional files 1 and 2). These genes and their products should be necessary for the maintenance of, or need to be repressed in, cells in a proliferative, non-differentiating ES cell state. Genes that mark developmental stages in the embryo, including Gbx2, Pitx2, Eomes, Amot, Dab2, Sox2, Fgf4, Nanog, Sall4, Fgf5, Timp1, Kdr, Cyp26a1, Dppa2 and Dppa4 were among this set, which is consistent with earlier studies [10,11]. Moreover, many of these differentially expressed genes have been previously found to be directly controlled by either Nanog [6], Sox2 [6] or polycombs [21].

**Figure 2:**
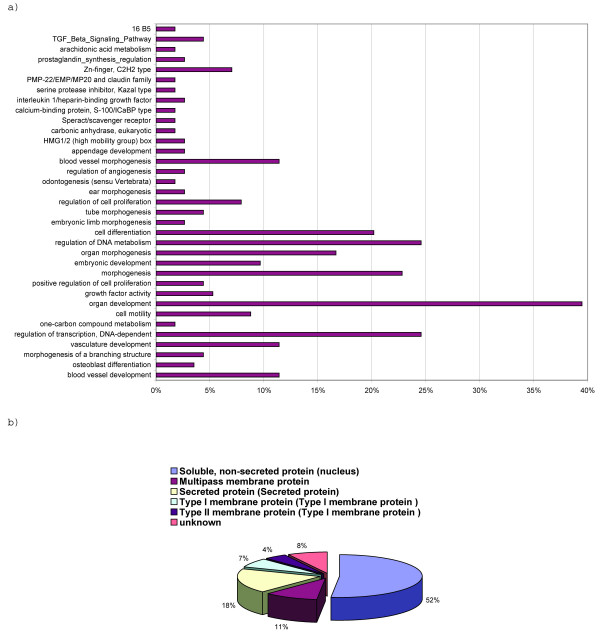
**Ontology classification**. Functional distribution (a) and subcellular location (b) for the set of genes that were differentially expressed between ES cells and differentiating cells.

The differentially expressed set of genes was assigned to functional categories, several of which were statistically overrepresented (*p *value < 0.005) in the gene set (Figure 2). Development-related genes, such as S100a6, Nrp1, Serpine1, Gbx2, and Pitx2 account for the largest portion (40%) of genes in G_stemness_. This was followed by genes for regulatory molecules, such as transcription factors for nucleobase, nucleoside, and nucleotide metabolism for rapid cellular proliferation, and by genes for proteins involved in intracellular signaling, cell surface receptors and ligands (30% of genes). In addition, genes associated with cell differentiation, embryonic development, organ morphogenesis, and the TGF-β signaling pathway were also found to be significantly overrepresented in G_stemness_. A comprehensive list of constituent genes in each overrepresented annotation term is provided as Additional file 3.

A large proportion of G_stemness _genes (52%) was classified as soluble intracellular proteins. Membrane proteins (type I/II and multi-pass) and soluble secreted proteins constituted only 21% and 18% of the entire set respectively. These latter two sets of genes form a group of genes whose protein products might be used as non-destructive markers of the differentiation state of embryonic stem cells.

### Diagnostic signature patterns using mRNA transcript expression levels

**Figure 3:**
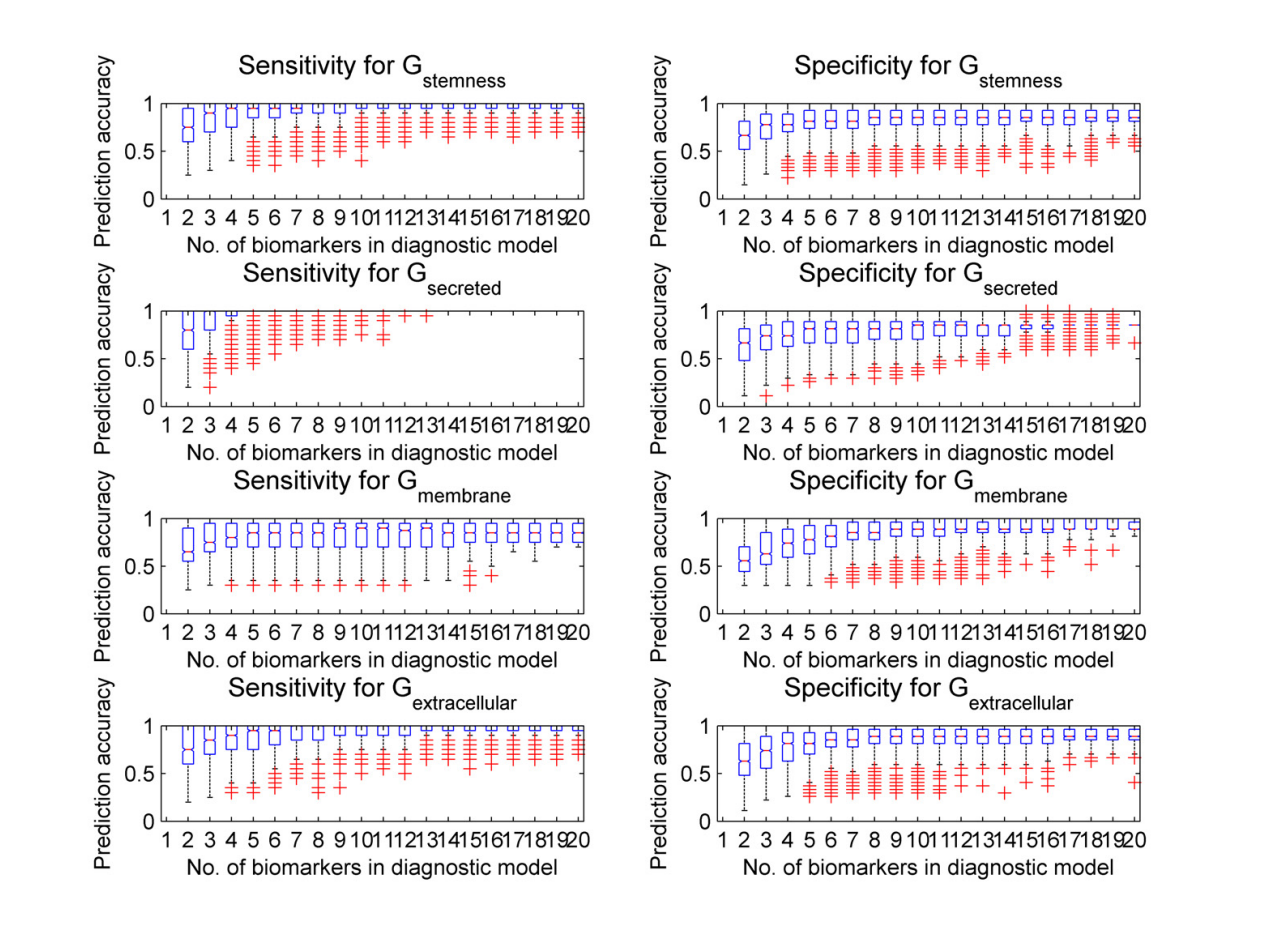
**Prediction accuracy**. Box plots of the accuracy (sensitivity or specificity) of the prediction of the differentiation state of embryonic stem cells using "n" (2 to 20) mRNA transcript levels as diagnostic signature patterns. The box has lines at the lower quartile (25^th^), median (50^th^), and upper (75^th^) quartile values. The whiskers are lines extending 1.5 × (inter-quartile ranges) from each end of the box with outliers ('+') marked. The box plot shows the average prediction accuracy (based on 500 random divisions of the sample set into training and test sub-sets) for the 30,000 diagnostic models created for each "n". The diagnostic models were built using G_stemness _(size = 114), G_secreted _proteins (size = 21), G_membrane _proteins (size = 25) and G_extracellular _proteins (size = 46) separately. Extracellular proteins include secreted and membrane proteins.

The Affymetrix data sets were used to determine diagnostic models intended to achieve high accuracy predictions of the stem cell state of the samples. For each n (2 to 20), 30,000 n-biomarker models were randomly selected from G_stemness_. Diagnostic signature patterns for these models were derived and evaluated based on 500 random divisions of the complete Affymetrix array set (47 microarrays) into training and test samples. Each test set consisted of 14 ES cell samples and 19 differentiating cell samples that were not used in the training process. To determine the minimum set of biomarkers that could achieve a high level of accuracy, the average performance of each n-biomarker diagnostic model on the 500 test data sets was plotted (Figure 3). Accuracy improved rapidly with the number of biomarkers included in the model up to 7, and reached a plateau when n exceeded 10. Because of the varying quality and predictive powers of individual biomarkers towards loss of pluripotency and initiation of differentiation, incorporating additional biomarkers into a diagnostic model may not increase the predictive power of that model. Some 5-biomarker diagnostic models that achieved higher prediction accuracy than 6-biomarker diagnostic models are shown in Additional file 4.

Among the four classes of stemness biomarkers used for constructing diagnostic models (Figure 3), those biomarker models that were built using selections of 2 to 20 genes from the class of 21 secreted proteins achieved the highest sensitivity with the least number of biomarkers. Certain biomarkers in that class, which are strongly related to developmental events or the maintenance of stem cells (e.g.: Igfbp4 [22], Bgn [23], fgf4 [24], fgf5 [25]), may increase the accuracy. However, the effectiveness of some of these models may be lower when used in other test situations as these models had lower specificity at higher values of n. This is probably due to the feeder layers of MEF cells also expressing 11 of these 21 biomarkers at high levels (see Additional file 1).

Even though some diagnostic models are able to achieve 100% accuracy in terms of their sensitivity and specificity, their predictive power in general may be questionable due to the expression of some of these genes by the feeder layers of MEF cells. Excluding these models, there were three 5-biomarker diagnostic models that achieved the highest prediction accuracy (Table 2). Using these models, ≥ 97% sensitivity and 100% specificity was achieved. This indicates that as few as five biomarkers may be sufficient to diagnose unknown test samples with high prediction accuracy.

**Table 2:** Optimum model details and test results. Expression levels in ES and differentiating (Diff) cells are used to make the signature pattern for the model. A 1 indicates that a gene is used in a model, 0 that it is not.

		**Expression in**		**Models**				
			
**Gene Symbol**	**Entrez ID**	**ES**	**Diff**	**1**	**2**	**3**	**all**	**Gene Name**
**Stmn2**	20257	405.51	159.89	1	1	1	1	stathmin-like 2
**Tcea3**	21401	175.47	67.29	1	1	1	1	transcription elongation factor A (SII), 3
**Igsf4a**	54725	63.81	158.42	1	1	0	1	immunoglobulin superfamily, member 4A
**Pitx2**	18741	87.00	348.96	1	1	1	1	paired-like homeodomain transcription factor 2
**Ramp2**	54409	93.57	247.39	1	0	1	1	receptor (calcitonin) activity modifying protein 2
**Tead2**	21677	218.96	617.77	0	1	1	1	TEA domain family member 2

	**Model Sensitivity (%)**				**Model Specificity (%)**			
	
**Data Set**	**1**	**2**	**3**	**All**	**1**	**2**	**3**	**All**

Affymetrix data sets (n = 47)	95.0	95.0	95.0	95.0	100.0	100.0	100.0	100.0
MPSS tpm count (n = 4)	100.0	100.0	100.0	100.0	100.0	100.0	100.0	100.0
NIA/Whitehead-Jaenisch cDNA arrays (n = 16)	100.0	100.0	100.0	100.0	NA	NA	NA	NA
Whole data set	97.4	97.4	97.4	97.4	100.0	100.0	100.0	100.0

These three optimum diagnostic models consisted of six stemness biomarkers, two of which, transcription elongation factor A (Tcea3) and stathmin-like 2 (Stmn2), are over-expressed in ES cells. The remaining four biomarkers, paired-like homeodomain transcription factor 2 (Pitx2), immunoglobulin superfamily, member 4A (Igsf4a), receptor (calcitonin) activity modifying protein 2 (Ramp2) and TEA domain family member 2 (Tead2), have reduced expression levels in ES cells but progressively increase their expression levels with differentiation. The results of diagnostic tests of the stem cell state of samples in the test data set, using these three 5-biomarker models, are summarized in Table 2.

**Figure 4:**
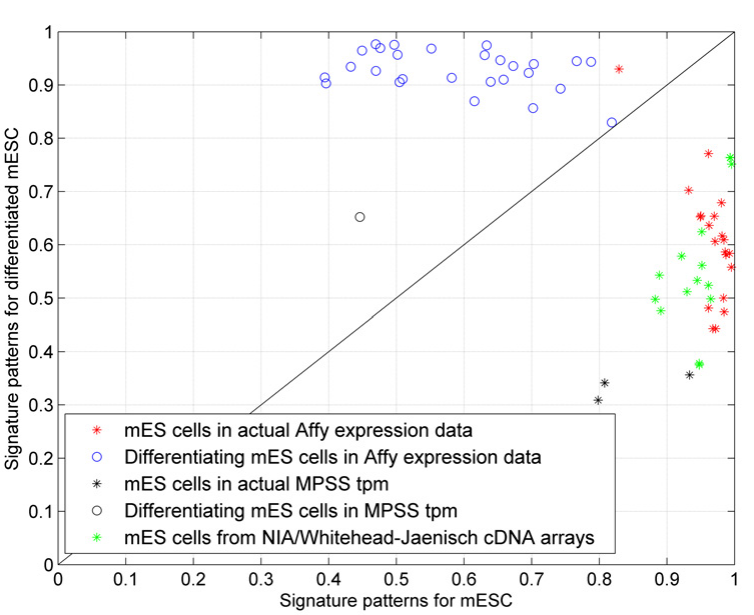
**Sample classification**. Projections of the biomarker expression levels in all ES cell and differentiating cell samples on the two diagnostic signature patterns of optimum model 1 are shown. One signature pattern correlates to the embryonic stem cell state and the other to the differentiating cell state.

Figure 4 shows the dot projection of each sample's expression levels for the 5-biomarkers of optimum model 1 on its signature patterns. The dot projections of expression levels on these two signature patterns are used to determine whether a sample is more closely related to differentiating cells or ES cells. Ideally, the magnitude of these projections should differ significantly from each other when the test sample is derived from a homogeneous cell population, with one projection being close to 1 and the other close to 0. However, due to the relative lack of synchrony during cell transition, most of the culture samples are usually derived from cell colonies that contain heterogeneous populations of differentiated, persistently pluripotent, and transiently pluripotent cell types [26,27]. In this case, the dot projections will deviate from the ideal case, and their values will provide information about the overall differentiation state for the majority of cells within the sample. Most of the test samples used here were found to be derived from relatively mixed populations of cell types.

Random permutation tests to evaluate the probability for obtaining the optimum diagnostic models by chance showed that only 9 of 1,000 randomised diagnostic models achieved ≥ 90% specificity and sensitivity, indicating that the probability of obtaining by chance a diagnostic model with the accuracy of the optimum model is less than 0.009.

## Discussion

### Determining biological significance of genes in the diagnostic signature patterns

By using studies of gene expression in embryonic stem cells and cells which have begun to differentiate, a panel of genes has been identified as biomarkers of the difference between the two states. These biomarkers could be used as a simple test of the differentiation state of a cell culture. A total of six biomarkers (Stmn2, Tcea3, Pitx2, Igsf4a, Ramp2, Tead2) were found to be most effective for this purpose. These genes were among the 10 genes that showed the most statistically significant differential expression between ES cells and differentiating cells.

Of these genes, stathmin-like 2 (Stmn2) was demonstrated to be a direct target of β-catenin/TCF-mediated transcription in hepatoma cells [28]. β-catenin/TCF is frequently activated during embryogenesis [29], consistent with its elevated expression in ES cell culture observed in the signature pattern. In addition, the stathmin family members were previously known to inhibit microtubule polymerization through their microtubule-destabilizing activity [30], and might inhibit cell differentiation which is known to involve a complete reorganization of the microtubule network [31].

Transcription elongation factor A (SII), 3 (Tcea3) encodes a protein that promotes read-through of transcriptional blocks by stimulating the nascent RNA cleavage activity of RNA polymerase II [32]. Tcea3 has also been identified as having ICM-specific expression [33]. Tcea3-/- mice demonstrated this gene to be indispensable for the self-renewal capability of hematopoietic stem cells [34].

The paired-like homeodomain transcription factor 2 (Pitx2) encodes a gene implicated in the establishment of left-right-handed asymmetry in mouse embryogenesis. It also regulates both morphogenesis and gene expression in developing extraocular muscles [35]. Together with other factors, it induces formation of many epithelial-derived organs, including bone [36], heart muscle [37] and hematopoietic [38] development. It would be expected that this gene will only be activated when ES cells begin differentiation, as reflected by the low expression level for this gene in ES cells.

The immunoglobulin superfamily, member 4A (Igsf4a) was first isolated as one of the genes preferentially expressed during neuronal differentiation of mouse embryonal carcinoma cells [39] and was later isolated in the mouse developing nervous system and epithelium of various organs [40]. Although its role as a mast-cell adhesion molecule for attachment to fibroblast cells has been clearly elucidated [41], little is known about its function during ES cell differentiation. Unlike the three biomarkers mentioned before, Igsf4a does not have a previously identified association with ES cell biology. However, its transcript level is consistently differentially expressed between ES cells and their differentiating progeny (*p *value ≈ 0).

Receptor activity modifying protein 2 (Ramp2) encodes a type I transmembrane protein modulator that, when in complex with calcitonin receptor-like receptor (CRLR), determines the ligand specificity of the CRLR on the cell surface. Together, they are known to play a key role in vasodilation and angiogenesis *via *the extracellular binding of adrenomedullin, which acts as a multifunctional peptide hormone [42]. Usually, the mRNA levels of CRLR and RAMP2 correlate strongly with adrenomedullin availability. Its expression level is low in ES cells and high when the cells start to differentiate.

Tead2, among all TEA domain family genes, is uniquely expressed in mouse embryos during the first week of early mammalian development [43]. This gene, which encodes a site-specific DNA binding protein, was previously confirmed to be expressed in several types of stem cells including embryonic stem cells [44]. Together with its transcriptional co-activator, YAP65, it can activate the expression of genes that serve different functions during cell differentiation and development. In agreement with previous results, the expression level for Tead2 in differentiating cells is almost 3-fold higher than its level in ES cells.

We have shown that the signature patterns derived here from a subset of the available training samples have high prediction accuracy when used on the remaining samples from which they were not derived. As these signature patterns were derived from ES cells differentiating following removal of LIF, the signature patterns might be affected if different differentiation conditions were used (e.g.: dimethyl sulfoxide (DMSO), retinoic acid (RA), ascorbic acid). Improvements in the prediction accuracy of the biomarker signature patterns by testing and refining them on a larger set of highly enriched cell lines will be possible in the future. Sampling bias might have affected the derivation of the patterns by treating ES cells and their differentiating counterparts as groups. The differentiation of ES cells includes the formation of distinct pluripotent cell populations that are characterized by their temporal expression of biomarkers [17] and this delineation could not be made here. Assignments provided by the original authors, which treated all pluripotent cell samples as a single discrete cellular entity of ES cells, were used. Similarly, the derivatives from ES cells, from 2–14 days, were treated as one group of differentiating cells.

Recently, Glover et al. [19] used a meta-analysis to investigate ES cell signature changes in three mouse ES cell lines. They discovered 88 genes with absolute changes in expression level that correlate well with decreasing frequency of ES cell pluripotency. Further validation, using Q-RT-PCR, of 22 of these genes led to a set of seven genes that showed rapid and consistent temporal down-regulation in expression across ES cell lines and differentiation protocols. In contrast to their method, which identifies ES cell signature change based on individual gene expression changes and bootstrap confidence values, this study evaluates ES cell signature changes based on a panel of biomarkers. Here the coordinated expression levels of a panel of both up- and down-regulated biomarkers were considered to jointly provide a signature pattern for diagnosis. In addition, instead of providing a set of parameter thresholds to be used in the definition of the ES cell signature change, several pairs of signature patterns consisting of five biomarkers (Table 2) were provided as a basis of comparison.

The diagnostic test developed here was applied to the dataset (E-MEXP-412) of Glover et al. [19]. Three differentiation conditions, (+LIF; +LIF+RA, and -LIF+DMSO), were used by those authors to study ES cell differentiation. The projection of biomarker expression levels in these three conditions onto the 5-biomarker (Stmn2, Tcea3, Igsf4a, Pitx2 and Ramp2) signature patterns derived here indicates that the signatures performed well under the first two conditions. This is consistent with Glover et al.'s finding that exposure to RA has the most rapid effect on ES cell differentiation. The response of ES cell differentiation to the third condition (-LIF+DMSO), however, was less prominent than that observed for RA. The projection of the signature patterns resulted in a differentiation status of cell population that is more ES cell-like (see Additional file 5). There are two possible reasons why the signature patterns did not detect the differentiated status of the DMSO-treated cells. First, DMSO may induce slower gene expression kinetics leading to the loss of cell pluripotency when compared with LIF removal and RA treatment [19]. Second, the signature patterns derived from ES cell differentiation following LIF removal may not be optimized to detect DMSO-induced cell differentiation. DMSO treatment induced expression of genes is associated with mesodermal differentiation [19] and was less rapid and homogeneous in differentiation to skeletal myoblasts than RA-induced ES cell differentiation to neuronal cells [45]. However, as there were only two biological replicates used by Glover et al. [19] for each differentiation protocol these results may be questionable.

### Going beyond mRNA, using secreted proteins from G_stemness _as diagnostic signature patterns

There is great interest in diagnosing the differentiation state of ES cells without the destruction of the cells. Three possible approaches to distinguish ES cells as they differentiate could be by using unique combinations of biomarkers that are either secreted extracellularly (G_secreted_), or are membrane bound proteins (G_membrane_) with extracellular domains (Type I/II and multipass membrane proteins). Their relative protein abundance could be assessed using techniques such as fluorescent-tagged signalling molecules or monoclonal antibodies that selectively adhere to these proteins. In total, 21 genes from G_stemness _are classified as soluble secreted proteins. Half of these are highly expressed in the mouse embryonic fibroblast cells used as feeder layers and therefore not useful as extracellular biomarkers. Signature patterns based on secreted proteins predict the cell state in the Affymetrix data sets accurately (sensitivity = 100%; specificity = 96.3%), however, they fail in other platforms (sensitivity_cDNA _< 40%) even when more biomarkers are included (see Additional file 6).

A second approach, using only membrane bound biomarkers G_membrane _as signature patterns, was also applied. Twenty of the 25 genes of this type in G_stemness _had low transcript levels in mouse embryonic fibroblast cells. Several n-biomarker diagnostic models (6 ≤ n ≤ 7) using these genes (e.g.: Ramp2, Trap1a, Cldn7, Gpc3, Mest, Slc29a1) were able to achieve high prediction accuracy across the three expression analysis platforms (specificity ~96.4%; sensitivity ~97.4%). A third approach used a combined set of secreted and membrane proteins (30 genes). Likewise, several n-biomarker diagnostic models (5 ≤ n ≤ 7) using these genes (e.g.: Fgf8, Spink3, Trap1a, Ptpns1, Mest, Car14) were able to achieve high prediction accuracy across the three expression analysis platforms. This overall prediction accuracy is comparable to that achieved by the optimum mRNA diagnostic model reported previously (see Additional file 7). However, due to the complex relationship between mRNA transcript levels and protein abundance, further experiments on these membrane bound biomarkers should be conducted to asses their usefulness towards detection of the differentiation status of ES cells.

## Conclusion

In this study, we demonstrated that the differentiation states of ES cells following LIF removal can be stratified efficiently using a set of diagnostic signature patterns consisting of only 5 "stemness" biomarkers. In contrast to other classification methods, this simple scheme derives diagnostic signature patterns that can be applied across different array platforms. It can be implemented in the laboratory to provide a convenient adjunct to conventional functional assays for determining the overall differentiation state of an ES cell colony following LIF removal.

## Methods

16 ES cell gene expression data sets from various laboratories were downloaded from the Gene Expresion Omnibus (GEO) [46] and ArrayExpress [47] or other sources, as noted (see Table 1). These data sets were created using several different technologies (Affymetrix Inc., NIA, Solexa and Lynx Therapeutics Inc.). Altogether, 39, 28 and 14 microarrays were available for embryonic stem cells, differentiating embryonic stem cells at various time points after LIF removal (≤ 14 days), and mouse embryonic fibroblast (MEF) cells, respectively.

**Table 1:** Details of the data sets used in this study.

	**No. of arrays**							
						
**Mouse cell lines**	**ES**	**Diff**	**MEF**	**Time points for Diff**	**Chip type**	**Induction/mESC phenotype**	**Ref.**	**Acc no.**
J1 ES Cells	3	3	0	5 days	Affy MOE430	LIF removal/-		
V6.5 (P18) ES Cells	3	15	0	6,12,18,24,36,48 hours,4,7,9,14 days	Affy MOE430	LIF removal/RT-PCR	[51]	GSE3231
R1 ES Cells	3	0	0	NA	Affy U74Av2	LIF removal/-	[52]	GSE2375
W9.5 ES Cells	2	6	0	1,2,3,4,5,6 days	Affy U74Av2	LIF removal/RT-PCR	[13]	E-MEXP-304
CCE ES Cells	2	0	0	NA	Affy U74Av2	LIF removal/-	[53]	Info^1^
C57B1/6 ES Cells	2	0	0	NA	Affy U74Av2	LIF removal/-	[44]	[54]
E14 ES Cells	2	0	2	NA	Affy U74Av2	LIF removal/-	[15]	[55]
R1 ES Cells	3	3	0	18,72 hours	Affy U74Av2	LIF removal/RT-PCR & Functional assays	[18]	[56]
DR4 Cells	0	0	3	NA	Affy MOE430	-	[51]	GSE3232
NIH-3T3 Cells	0	0	4	NA	Affy U74Av2	-	[57]	GSE2192
Primary MEFs from wild-type C57Bl/6 13.5 day embryos	0	0	2	NA	Affy MOE430	-	[58]	GSE2684
T1/2 fibroblasts Cells	0	0	3	NA	Affy MOE430	-	[51]	GSE3236
E14 ES Cells	3	1	0	4 days	MPSS data sets – two sources	LIF removal/-	[51]	GSE1581; [59]
CCE ES cells	10	0	0	NA	Whitehead-Jaenisch mouse operon 32k v4.1	LIF removal/-	[51]	E-MEXP-501
R1 ES Cells	3	0	0	NA	NIA Agilent Mouse 44K Microarray v2.1	LIF removal/-	[52]	E-MNIA-66
R1 ES Cells	3	0	0	NA	NIA mouse 22k Ver1	LIF removal/-	[13]	E-MNIA-73

Array totals	39	28	14					

Info^1^: through personal communication;

### Data pre-processing and identification of differentially expressed biomarkers (G_stemness_)

Gene probes that were common to all Affymetrix data sets were first identified based on the manufacturers' annotations. The probes from cDNA and MPSS studies were later mapped to Affymetrix probes using common gene names. This gave approximately 10,000 well-characterized mouse genes that were used for further analysis. Raw CEL files, were normalised at the PM and MM probe level, by global scaling. Gene expression levels were obtained using the Li-Wong model-based gene expression indices using dChip software [48]. All arrays were used to develop the model, and a smoothing spline normalization method was applied. Expression values below 10 were set to 10.

The ES cell and differentiating cell arrays generated from Affymetrix platforms were separated into two pools to determine expression differences between them. MEF cell arrays were excluded from this analysis. Genes were considered to be differentially expressed between the two samples (denoted G_stemness_) if they fulfilled the three following criteria:

1) the gene was called absent in all arrays of one sample type, but present or marginal in all arrays of the other type; or

2) the 90% lower confidence bound (LCB) of the fold change between the two sample types is above 2; and

3) the statistical significance of the detected fold change was ≤ 0.005 [48].

Genes that were determined to be differentially expressed between the two states were assigned to general functional categories using Gene Ontology (GO) terms [49]. A hypergeometric distribution test was used to assess whether a given functional annotation was over-represented in the set of differentially expressed genes. Subcellular localization details of the differentially expressed genes were extracted from the curated LOCATE database of the subcellular localizations of the mouse proteome [50]. Based on this information, G_stemness _was separated into 5 sub-classes (soluble non-secreted proteins; type I membrane proteins; type II membrane proteins, multi-pass membrane proteins; and secreted extracellular proteins). Among them, the latter four were referred to as extracellular proteins.

### Diagnostic signature patterns for ES cells and their differentiating counterparts

Biomarker diagnostic models were built by taking the average expression level for a gene or biomarker in ES cell samples and in differentiating cell samples. For each cell type, an n-biomarker model is constructed from the averaged expression levels of "n" biomarkers or genes. These form an n-dimensional "signature" vector for each cell type which is then normalized to unit length. Only the Affymetrix chip data (20 ES cell samples and 27 differentiating cell samples) were used for this process. For cross validation purposes, average expression levels were taken from a randomly selected 30% of the total samples for each cell type (i.e. 6 and 8 samples from ES cell and differentiating cell groups, respectively, were used as the training set) and the remaining samples were used as the test set. The last step was repeated 500 times to avoid biased diagnostic signature patterns (see Error rate estimation for n-biomarker diagnostic models below).

### Determination of differentiation cell state based on diagnostic signature patterns

**Figure 1:**
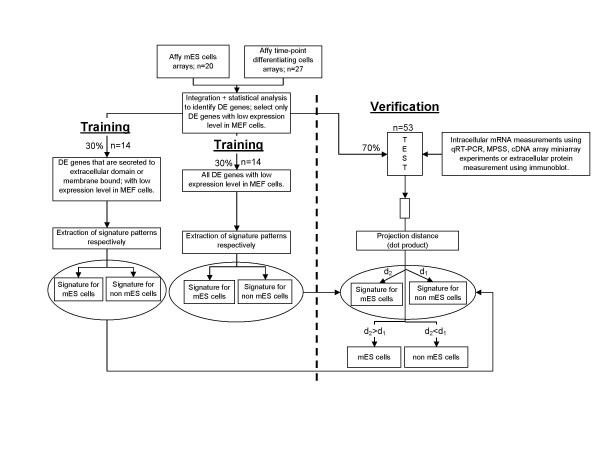
**Work flow**. The proposed mRNA molecular diagnostic strategy used to stratify the differentiation state of mouse embryonic stem cells. (DE = differentially expressed).

Samples from the test or other data set are assessed, using an n-biomarker diagnostic model, for their stem cell state by constructing a normalised vector of the expression levels of the n-biomarker genes in the sample to be tested. The scalar projections of this test vector with the two signature vectors (one for each cell type as defined above) are calculated and the test sample is defined as belonging to the class or cell type to which it has the larger projection. The schematic illustration of the diagnostic procedure is shown in Figure 1.

### Error rate estimation for n-biomarker diagnostic models

To obtain an unbiased estimation of the error rate associated with the training samples selected, 500 random selections of 30% of the samples within each cell type were used to build n-biomarker diagnostic models. The accuracy of the n-biomarker diagnostic model was assessed using the test data set based on its sensitivity (true predictions of ES cell state/number of test ES cell samples) and specificity (true predictions of "not ES cell" state/number of test "not ES cell" samples). An average error rate for the n-biomarker diagnostic model could then be calculated.

Given the size of G_stemness _(114 genes), the number of n-biomarker diagnostic models that could be built is intractably large. The number of possible 6-gene models is O(10^10^). In this study, 30,000 diagnostic models, randomly selected for each n (ranging from 2 to 20), were assessed. Optimum n-biomarker diagnostic models were selected based on the following criteria:

1) the highest sensitivity and specificity;

2) the expression levels of all n biomarkers in the model are low in MEF.

To investigate whether these models could arise by chance, 1000 random permutations of the cell samples were made to give randomized expression vectors for the biomarkers in each model, and the procedure above was used to estimate prediction accuracy of the randomized diagnostic. The probability that the accuracy of randomized diagnostic models exceeds 90% in both sensitivity and specificity was computed.

## Authors' contributions

DYLY and DKS acquired the array data sets, participated in the design, performed microarray analysis and drafted the manuscript. XWZ participated in the design, discussion, interpretation and revision of the results and manuscript. JH participated in the revision of the manuscript. All authors read and approved the final manuscript.

## Supplementary Material

Additional file 1 Stemness genes (G_stemness_). The complete list of genes identifed to be significantly differentially expressed between ES cells and differentiating counterparts. Click here for file

Additional file 2 Gene expression profiles. The expression levels of the set of 114 differentially expressed genes (G_stemness_) in mouse embryonic fibroblast cells, embryonic stem cells and their differentiating counterparts are shown. Genes were clustered based on the correlation of their expression profiles as implemented in dChip. Cell samples are represented in columns; genes in rows. The known regulators (Nanog, Oct-4 and polycombs) are indicated. The level of gene expression is colour-coded from blue (low) to red (high). Click here for file

Additional file 3 Overrepresented annotation for stemness genes. The differentially expressed set of genes was assigned to functional categories, several of which were statistically overrepresented (*p *value < 0.005) in the gene set. This file provides a comprehensive list of constituent genes in each overrepresented annotation term. Click here for file

Additional file 4 Optimum diagnostic models. The 5-gene optimum diagnostic models found in this study. Because of the varying quality and predictive powers of individual biomarkers towards loss of pluripotency and initiation of differentiation, incorporating additional biomarkers into a diagnostic model may not increase the predictive power of that model. Some 5-biomarker diagnostic models can achieve higher prediction accuracy than 6-biomarker diagnostic models as show in this table. Click here for file

Additional file 5 Additional sample classification (PLOS comp biol. 2006 2:e158 – MEXP-412 dataset). The diagnostic test developed here was applied to the dataset (E-MEXP-412) of Glover et al. [19]. Three differentiation conditions, (+LIF; +LIF+RA, and -LIF+DMSO), were used by those authors to study ES cell differentiation. The projection of biomarker expression levels in these three conditions onto the 5-biomarker (Stmn2, Tcea3, Igsf4a, Pitx2 and Ramp2) signature patterns derived here indicates that the signatures performed well under the first two conditions. This is consistent with Glover et al.'s finding that exposure to RA has the most rapid effect on ES cell differentiation. The response of ES cell differentiation to the third condition (-LIF+DMSO), however, was less prominent than that observed for RA. The projection of the signature patterns resulted in a differentiation status of cell population that is more ES cell-like. Click here for file

Additional file 6 Example of diagnostic model based on secreted proteins not expressed in MEF. In total, 21 genes from G_stemness _are classified as soluble secreted proteins. Half of these are highly expressed in the mouse embryonic fibroblast cells used as feeder layers and therefore not useful as extracellular biomarkers. Signature patterns based on secreted proteins predict the cell state in the Affymetrix data sets accurately (sensitivity = 100%; specificity = 96.3%), however, they fail in other platforms (sensitivity_cDNA _< 40%) even when more biomarkers are included. Click here for file

Additional file 7 Example of diagnostic model based on membrane proteins not expressed in MEF. A combined set of secreted and membrane proteins (30 genes) were used to construct diagnostic models. Several n-biomarker diagnostic models (5 ≤ n ≤ 7) using these genes (e.g.: Fgf8, Spink3, Trap1a, Ptpns1, Mest, Car14) were able to achieve high prediction accuracy across the three expression analysis platforms. This overall prediction accuracy is comparable to that achieved by the optimum mRNA diagnostic model reported previously. Click here for file
