# Supplementary figures and images for: Using biomarker signature patterns for an mRNA molecular diagnostic of mouse embryonic stem cell differentiation state
Source: BMC Genomics. 2007 Jul 3;8:210. doi: 10.1186/1471-2164-8-210 (PMC1931595; doi:10.1186/1471-2164-8-210)

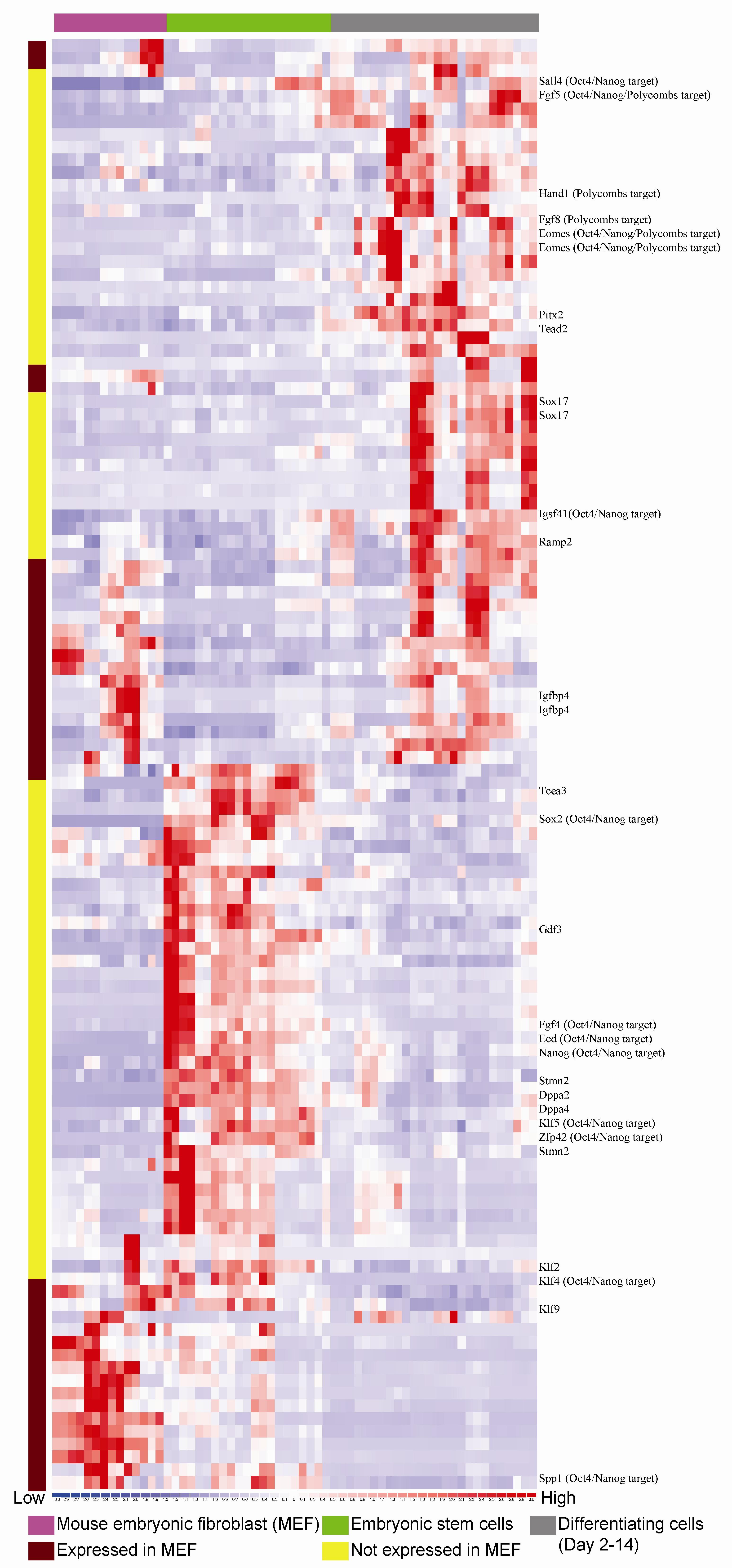

Supplement: Additional file 2 — Gene expression profiles. The expression levels of the set of 114 differentially expressed genes (Gstemness) in mouse embryonic fibroblast cells, embryonic stem cells and their differentiating counterparts are shown. Genes were clustered based on the correlation of their expression profiles as implemented in dChip. Cell samples are represented in columns; genes in rows. The known regulators (Nanog, Oct-4 and polycombs) are indicated. The level of gene expression is colour-coded from blue (low) to red (high). [file 1471-2164-8-210-S2.jpeg]
