# Supplementary material for: Using biomarker signature patterns for an mRNA molecular diagnostic of mouse embryonic stem cell differentiation state
Source: BMC Genomics. 2007 Jul 3;8:210. doi: 10.1186/1471-2164-8-210 (PMC1931595; doi:10.1186/1471-2164-8-210)
Supplement: Additional file 5 — Additional sample classification (PLOS comp biol. 2006 2:e158 – MEXP-412 dataset). The diagnostic test developed here was applied to the dataset (E-MEXP-412) of Glover et al. [19]. Three differentiation conditions, (+LIF; +LIF+RA, and -LIF+DMSO), were used by those authors to study ES cell differentiation. The projection of biomarker expression levels in these three conditions onto the 5-biomarker (Stmn2, Tcea3, Igsf4a, Pitx2 and Ramp2) signature patterns derived here indicates that the signatures performed well under the first two conditions. This is consistent with Glover et al.'s finding that exposure to RA has the most rapid effect on ES cell differentiation. The response of ES cell differentiation to the third condition (-LIF+DMSO), however, was less prominent than that observed for RA. The projection of the signature patterns resulted in a differentiation status of cell population that is more ES cell-like. [file 1471-2164-8-210-S5.doc]

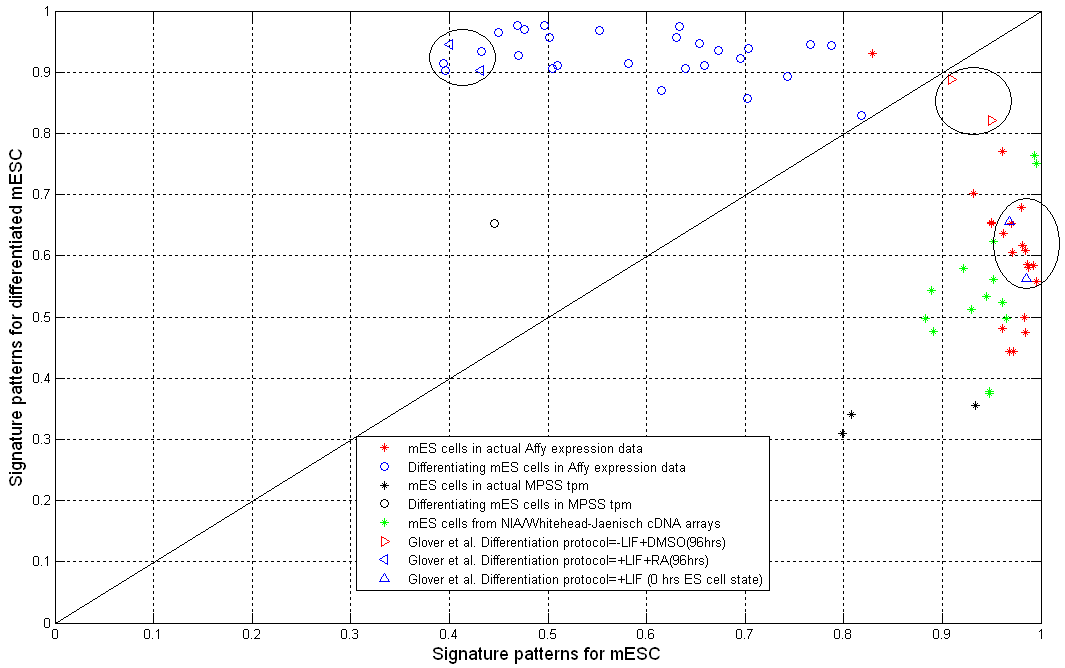


**Supplementary Figure 1 : Additional Sample Classification**

Projections of the biomarker expression levels in all ES cell and differentiating cell samples and the data of Glover et al (Plos Comp Biol. 2006 2:e158 - M-EXP-412) on the two diagnostic signature patterns of optimum model 1 are shown. One signature pattern correlates to the embryonic stem cell state and the other to the differentiating cell state.
